# ^106^Ruthenium eye plaque brachytherapy in the management of medium sized uveal melanoma

**DOI:** 10.1186/s13014-020-01621-4

**Published:** 2020-07-29

**Authors:** Ping Jiang, Konstantine Purtskhvanidze, Gerit Kandzia, Dirk Neumann, Ulf Luetzen, Frank-André Siebert, Johann Roider, Juergen Dunst

**Affiliations:** 1 University Clinic for Medical Radiation Physics, Medical Campus Pius-Hospital, Carl von Ossietzky University, Georgstrasse 12, Oldenburg, Germany; 2 Department of Ophthalmology, University Clinic Schleswig-Holstein, Campus Kiel, Germany; 3 Department of Radiation Oncology, University Clinic Schleswig-Holstein, Campus Kiel, Germany; 4 Department of Nuclear Medicine, University Clinic Schleswig-Holstein, Campus Kiel, Germany

**Keywords:** Ruthenium, Radiotherapy, Plaque brachytherapy

## Abstract

**Background:**

To evaluate ^106^Ruthenium Brachytherapy in management of medium sized uveal melanoma, with emphasis on 5-year outcome and toxicity.

**Methods:**

From April 2007 to October 2015, 39 patients with medium sized uveal melanoma were treated with 106 Ru eye plaques brachytherapy. At the time of diagnosis, the mean tumor depth was 3.7 mm (±SD:1.6 mm). The mean dose at the tumor apex was 141.4 Gy (± SD: 12.1 Gy) and 557.7 Gy (± SD: 257.3 Gy) to the sclera.

**Results:**

Mean follow-up was 69.5 months (± SD: 53.8 months). Thirty-four patients (87.1%) remained free of recurrence. Twenty-six patients (66.7%) demonstrated a complete tumor regression after a median period of 12 months (3–60 mon.). By the final examination, the visual acuity of 26 patients (66.7%) was better than 20/200, and 12 patients (30.7%) had a visual acuity better than 20/40.

Retinopathy was detected in 11 patients (28.2%). After treatments only one patient (5.1%) had active vascular changes by the last examination. Moderate optic neuropathy was observed in 4 patients (10.3%). Cataract development was diagnosed in 21 patients (53.8%), and 16 patients (41%) had bilateral cataract development.

Special emphasis was made on patients with larger tumors. Twelve out of the 39 patients had a tumor with a depth of 5 mm or more. There was no significant difference in local control or in side effects between both groups observed.

**Conclusions:**

Our study proved ^106^Ru -brachytherapy to be an excellent treatment option with regard to tumor control and preservation of the visual acuity in well-selected patients. Our data suggested that this treatment is also suitable for tumors with a depth of more than 5 mm.

## Background

Plaque brachytherapy has been well accepted for decades as an efficient approach for treatment of medium sized uveal melanoma. The largest prospective randomized trial with over 1300 patients, the Collaborative Ocular Melanoma Study (COMS), found no prognostic benefits in patients who had undergone enucleation in comparison with plaque brachytherapy [1]. As an organ sparing treatment, plaque brachytherapy has psychological benefits and improves quality of life (QoL) of the patients substantially. However, the optimization strategies of plaque brachytherapy are under discussion. Different isotopes such as ^125^I, ^103^Pd, ^106^Ru, ^90^Sr and ^131^Cs were used for plaque brachytherapy and the treatment concepts varied considerably between different institutes.

As a beta emitter, the radiation gradient surrounding ^106^ Ru is steeper than the gradient surrounding a gamma emitter like ^125^I or ^103^Pd. The radiation to distal critical eye structures such as macula and optic nerve is therefore decreased during treatment with ^106^Ru plaque therapy. Traditionally, the use of ^106^Ru plaque therapy is preferred in Europe and Asia. This treatment was commonly used to treat uveal melanomas with tumor depth of up to 5 mm in the world [2, 3].

The treatment strategy of ^106^ Ru plaque brachytherapy has been established for more than 20 years in our clinic. We recently reviewed our treatments of ^106^Ru brachytherapy in patients with medium sized uveal melanomas from 2007 to 2015 with emphasis on 5-year outcome and toxicity.

## Methods

The diagnosis, localization and tumor-size measurement of uveal melanoma were performed using transilumination, fundoscopy, ultrasound, and other presurgical ophthalmologic examinations. Patients whose visual acuity could no longer be saved underwent an enucleation. Patients with small or medium sized uveal melanoma up to 8 mm deep were treated with ^106^Ru eye plaques brachytherapy after ruling out contact with the optic disc or macular, while those in the same group but with optic disc contact or macular involvement were referred for proton beam therapy. Other treatment strategies, such as endoresection, transpupillary thermotherapy, photodynamic therapy or chemotherapy were only considered in cases of recurrence or metastasis.

### Brachytherapy treatment

Patients remained hospitalized in appropriate rooms during the brachytherapy treatment period. General anesthesia was administered during the surgical procedures. Ocular muscles would be relocated when they interfered with plaque position, and then stitched back in the original position after removing the plaque.

Three types of ^106^ Ru eye applicators were used in our institute (Eckert & Ziegler BEBIG GmbH, Berlin, Germany): CCA, CIB, CCB. To ensure that a safety margin of at least 2 mm was added on all margins of the gross tumor volume (GTV), the chosen plaque was 4 mm bigger than the largest tumor in diameter.

Two patients with large tumor size, 17x12x6 mm and 18 × 5.1 × 3.8 mm in diameter, respectively, were treated with two applicators, CCB and CIB, sequentially. After the prescribed dose of the first implanted applicator (CCB) was attained, the second applicator (CIB) was attached near the CCB applicator before its removal.

Treatment planning was based on the source certificates of the eye plaques. In particular, the reference dose rate at 2 mm depth and depth dose rate curves were used in an in-house Excel dose calculation sheet. Using this calculation sheet the treatment time for the plaques are computed considering the actual dose rate, depth dose rate curves, and prescription dose at 2 mm of the sources. The source certificates state an uncertainty of dose rate at reference point of ±20% (k = 2). Corrections for tumor asymmetry or variations in concentricity of the sources were not applied [4].

The fundoscopy and ultrasound were used to localize the tumor. Transillumination was used to locate tumor margins. The implant location enclosed the tumor and the safety margin was marked at first with a pen on the sclera. The Plaque was sewed to the marked position. Uncertainties in positioning were taking into account by using the 2 mm safety margin around the GTV. To ensure that a safety margin of at least 2 mm was added on all margins of the GTV, the chosen plaque diameter was 4 mm more than the largest tumor diameter.

The treatment planning for the two patients with two applicators were conducted as a standard treatment plan. A consideration of partial dose overlap in the treatment planning process was not possible. Nevertheless, it was endeavored at the implanting to produce little geometrical and thus dosimetric overlap only.

A pre-treatment plan was calculated with a prescribed dose of 150 Gy at the apex of the tumor, taking into account that a cumulated dose of not more than 1200 Gy should be delivered to the sclera, otherwise a clinical decision must be made to treat the tumor. A depth of 1 mm corresponding to the sclera thickness was added to the real depth measured. After the treatment, the applied plan was calculated and documented.

### Treatment evaluation

Follow-up examinations were performed at 3-month intervals for the first year, then twice a year thereafter up to 5 years after treatment. Sixteen patients had further examinations annually.

Tumor response was evaluated using fundoscopy and ultrasound. Metastases were investigated routinely, and additional use of imaging methods was applied in symptomatic patients. Tumor size and visual acuity were documented on each follow up examination. Glaucoma was defined as intraocular pressure (IOP) ≥21 mm Hg. Recurrence was defined as the tumor growth more than 1 mm in any directions. Hemorrhagic or exudative tumor enlargement was observed for regression prior to being considered growth.

All statistical analysis was performed with SPSS v24.0 (SPSS, Chicago, IL). The numeric variables are expressed as median and range or mean with standard deviation. The rates of tumor response and the adverse events are shown as percentages. Significance of the differences in the tumor response and the adverse events between subgroups of patients were assessed by χ2 tests. *p* < 0.05 was considered statistically significant.

## Results

### Patient characteristics

From April 2007 through October 2015, 39 patients (26 females and 13 males) were treated with ^106^Ru eye plaque brachytherapy in our institute. The median age at diagnosis was 71.1 years (Range: 37–88 years). At the time of diagnosis, the mean tumor depth was 3.7 mm (±SD:1.6 mm). For patients with a tumor depth less than 2 mm, documented growth before intervention was a prerequisite. The mean distance to papilla and macula was 6.6 mm (±SD: 2.8 mm), resp. 3.8 mm (±SD: 2.7 mm). Two patients had previously received ^106^Ru brachytherapy with 140 Gy and 150 Gy, respectively.

Initially 38 patients (97.4%) had a visual acuity better than 20/200. Twenty-three patients (59.0%) had visual acuity better than 20/40. Mean dose at the apex of the tumor was 141.4.0 Gy (± SD: 12.1 Gy). The mean sclera dose was 557.7 Gy (± SD: 257.3 Gy) (Table 1).

**Table 1:** Patient characteristics

Patient variables		Median	Mean	No. of patients	%
Age (years)		71.1 (Range: 37–88)			
Male				13	33.3%
Female				26	66.7%
Visual acuity > 20/40				23	59.0%
20/40–20/200				15	38.5%
< 20/200				1	2.6%
*Tumor depth (apical height mm)*			3.7 (±SD:1.6)		
< 2.5				9	23.1%
2.5–5.0				18	46.2%
5.0–8.9				12	34.3%
Minimum distance to the papilla (mm)			6.6 (±SD:2.8)		
Minimum distance to the macula (mm)			*3.8* (±*SD:2.7)*		
Plaque type	CCB			20	51.3%
	CIB			6	15.4%
	CCA			15	38.5%
Administered dose (Gy)			141.4 (±SD:12.1)		
Treatment time (hours)			112.8 (±SD: 73.7)		
Scleral dose (Gy)		444.0 (Range: 185–1271)	557.7 (±SD:257.3)		

### Tumor response and visual acuity

The mean length of follow-up was 69.5 months (± SD: 53.8 months). Thirty-four cases (87.1%) remained free of recurrence. Five patients (12.8%) developed metastatic disease. Three patients died during the follow-up period.

In 26 patients (66.7%) a total regression was achieved after a median period of 12 months (Range: 3–60 mon.). By the final examination, 26 patients (66.7%) had visual acuity better than 20/200. Twelve of 23 patients (52.2%), whose initial visual acuity was better than 20/40, preserved it after the treatment (Table 2).

**Table 2:** Treatment results in tumors with a depth of ≥5 mm versus < 5 mm

Tumor depth (apical height)	< 5 mm		≥5 mm		*p*-Value
Patient number	27		12		
Tumor recurrence	2 (7.4%)		3 (25%)		0.129
Retinopathy	9 (33.3%)		2 (16.7%)		0.286
Optic neuropathy	3 (11.1%)		1 (8.3%)		0.792
Cataract of treated eye	15 (55.6%)		6 (50%)		0.748
Cataract both sides	12 (44.4%)		4 (33.3%)		0.515
Hemorrhage	1 (3.7%)		0		0.499
Metastasis	1 (3.7%)		4 (33.3%)		0.011
Visual acuity	initial	posttreatment	initial	posttreatment	
> 20/40	15 (55.5%)	10 (37.0%)	8 (66.7%)	2 (16.7%)	
20/40–20/200	11 (40.7%)	8 (29.6%)	4 (33.3%)	6 (50%)	
< 20/200	1 (3.7%)	9 (33.3%)	0	4 (33.3%)	

### Early and late treatment-related side effects

Retinopathy was detected in 11 patients (28.2%) after a median period of 18 months (Range: 3–24 months). The changes due to retinopathy responded well to laser therapy. By the last examination, only 1 patient had active vascular changes.

Moderate and mild optic neuropathy was observed in 4 patients (10.3%), and 2 patients (5.1%), respectively. Cataract development was diagnosed in 21 patients (53.8%), and 16 of them (41.2%) had bilateral cataract development. Excluding three patients with incomplete documentation, no glaucoma patient was found.

### Impact of tumor depth on treatment results

Special emphasis was made on patients with larger tumors. Twelve of the 39 patients had a tumor with a depth of 5 mm or more. We compared the treatment results in tumors with depth ≥ 5 mm and < 5 mm (Table 2). Although a better local control rate (92.6%) was observed in tumors < 5 mm, comparing to 75% in tumors ≥5 mm, the difference is not statistically significant (*p* = 0.129). There was no difference observed in side effects between both groups (retinopathy 16.7% vs. 33.3%, optic neuropathy 8.3% vs. 11.1%). As expected, larger tumors were significantly correlated with the risk of the metastases.

## Discussion

Successful treatment of uveal melanomas with preservation of the eye and visual function is one of the great achievements in oncology in the twentieth century. Plaque brachytherapy as well as external beam radiotherapy with charged particles therapy (CPT) can achieve very high rates of local control and play an important role in eye preservation. However, it is still challenging to optimize radiation treatment especially in larger tumors with regard to tumor control, visual outcome and treatment related side effects.

In this study, the 5-year local control rate of uveal melanomas after ^106^Ru eye plaques brachytherapy reached 87.1%, which is consistent with the local control rate of 89.7% reported by COMS report 19 [5]. However, the local control failure of COMS study was defined as the progression of the height of the tumor in 25%. When ^125^I plaque therapy was applied, the rates of tumor controlling varied from 88.2 to 94% in diverse studies [6–8]. The 5-year local recurrence rate of CPT was around 5% [9–11]. Confounding factors that affect treatment outcomes among different studies include the selection of patients, tumor characteristics and the definition of the recurrence rate. In our study, the 5-year tumor control rate close to 90% is a good result and confirming the efficiency of ^106^Ru eye plaque brachytherapy for treatment of uveal melanomas.

Intraocular side effects observed in our study were rare. 28.2% patients developed retinopathy. However only 2.6% patients had active vascular changes after the treatment. 10.3% patients had optic neuropathy and 12.8% patients developed unilateral cataract. No enucleation due to complications was necessary. Recently studies indicated that ^106^Ru eye plaque brachytherapy generally causes fewer late complications than ^125^I therapy [8, 12]. A meta-analysis of CPT involving 7500 patients in 28 studies also revealed a low ocular toxicity similar to our study. The pooled rate of radiation retinopathy was 0.28 (95% CI, 0.15–0.41), 0.34 for cataract formation (95% CI, 0.15–0.53), 0.21 for optic neuropathy (95% CI, 0.04–0.38), and 0.02 for enucleation due to complications (95% CI, 0.00–0.04) [13] .

In a study of proton beam therapy involving 2413 patients, Desjardins and coworkers reported that 42% of patients preserved visual acuity of more than 20/200 after a median follow-up of 98 months [14]. In this study, the median size of treated tumor was 4.7 mm in depth and additional treatment to improve final visual results were used. The 3-year visual acuity of more than 20/200 was reported from 45 to 73% in several studies after ^125^I plaque brachytherapy [15–17]. In our study, the preservation of useful vision (more than 20/200) was achieved in 66.7% of patients after a median follow-up of 61.5 months, and 52.2% of patients (total number of 12) kept their initial visual acuity better than 20/40 after the treatment.

Brachytherapy offers a variety of practical advantages. It is a cost-effective treatment technique and can be offered in a large number of radiotherapy centers. During brachytherapy, the plaque is attached to the eye wall so that the intrafraction motion is reduced and neglectable, which is an important advantage compared to external beam techniques. Moreover, plaque brachytherapy results in less anterior segment complications due to the short distance between the brachytherapy plaque and the target. Unlike charged particle therapy, where patient’s cooperation and comprehension are necessary, plaque brachytherapy can be carried out without these pre-requisites. The good visual outcome and low intraocular side effects revealed by our study prove that ^106^Ru brachytherapy is an excellent treatment option in well-selected patients.

Barker and coworkers reported a high tumor recurrence rate by ^106^Ru treatments with 75.5 Gy radiation doses delivered to the tumor apex [18]. Barker also suggested that the brachytherapy-planning protocols used for ^125^I, for example COMS, were not sufficient for ^106^Ru plaque brachytherapy due to the dosimetric difference between ^125^I und ^106^Ru. Three large ^106^Ru treatment series with a mean dose of 100 Gy to the apex reported 5-year local control rates at 78, 82, and 84% respectively [19–21]. Another prospective study of ^106^Ru brachytherapy on 450 patients reported that a 5-year local control rate of 97.9% could be achieved if a minimal-scleral radiation dose of 300–400 Gy was used [22]. It was estimated that 96% of those patients received more than 100 Gy apex dose, while 73% of them received more than 125 Gy and 54% with more than 150 Gy [22]. Overall, more studies are needed to determine the optimal treatment radiation dose for ^106^Ru brachytherapy. What we reported in this study showed that our protocol with up to 150 Gy at the tumor apex was feasible, effective and safe.

In North America, ^106^Ru brachytherapy is normally considered for tumors with a depth of less than 5 mm. Our results showed that treatment of uveal melanomas with an apex depth more than 5 mm (up to 8 mm in this study) with ^106^Ru brachytherapy was also possible. There was no correlation between tumor depths and either local control rates or late toxicity in our study. However, our study showed that large tumors were correlated significantly with higher metastasis rate. As a one-shot gentle treatment, ^106^Ru plaque brachytherapy provides good local control of the tumor and protects the visual acuity. Overall, it improves the QoL of the patients.

Like other studies involving ^106^Ru brachytherapy, our study has some limitations. First of all, the sample size is small and the outcome is based on retrospective analysis. Secondly, there have been changes in the whole treatment concept over the past years; the use of additional therapies like transpupillary thermotherapy, photodynamic therapy, and other therapy modalities varied over time and might have had an impact on the results. Moreover, direct comparisons of ^106^Ru brachytherapy with other therapies in randomized trials are lacking.

## Conclusions

The major findings in our study were the high local control rate with ^106^Ru brachytherapy even in tumors with a depth of more than 5 mm and the overall low acute and long-term toxicities. Despite the limited number of patients, these results prove ^106^Ru brachytherapy to be an excellent treatment option in clinical practice for uveal melanoma and should be promoted in well-selected patients. The treatment is also suitable for tumors with a depth of more than 5 mm.

## Data Availability

The datasets used and/or analyzed during the current study are available from the corresponding author on reasonable request.

## Abbreviations

COMS: Collaborative Ocular Melanoma Study
^106^Ru: ^106^ruthenium
^125^I: ^125^iodine
^103^Pd: ^103^palladium
^90^Sr: ^90^strontium
^131^Cs: ^131^cesium
CPT: Charged particles therapy
